# Multimodal Exercises Adjunct to Virtual Reality in Acromioclavicular Joint Sprain Rehabilitation: A Case Report

**DOI:** 10.7759/cureus.66599

**Published:** 2024-08-10

**Authors:** Palash Satone, Swapnil U Ramteke, Pratik R Jaiswal

**Affiliations:** 1 Sports Physiotherapy, Ravi Nair Physiotherapy College, Datta Meghe Institute of Higher Education & Research, Wardha, IND

**Keywords:** acromioclavicular joint, sprain, virtual reality (vr), rehabilitation, acromioclavicular injuries

## Abstract

Acromioclavicular joint (AC) sprains are common, usually resulting from a fall on the corner of the shoulder or, less often, an outstretched arm. In this report, we discussed the assessment and physiotherapy protocol along with virtual reality (VR) training of a 21-year-old male state-level kabaddi player who complained of pain in his left shoulder following a history of fall on his left shoulder while playing. This study highlights clinical assessment, diagnostic assessment, therapeutic intervention, and outcomes for patients with a grade II AC sprain. Pain, range of motion (ROM), and muscle strength were clinically assessed. The patient was managed with cryotherapy, movement with mobilization (MWM), rigid taping, ROM exercises, VR training, and muscle strengthening. The results of the study concluded that our conventional physical therapy along with MWM adjunct to VR facilitates the patient’s functional recovery.

## Introduction

The articulation of the acromion with the lateral end of the clavicle forms the acromioclavicular (AC) joint, which extends anteriorly to the scapula, providing both mobility and stability to the shoulder joint [1]. AC joint injuries, such as dislocations and sprains, are common events in the shoulder. Injury to the ligament(s) of the AC joint is caused by either direct or indirect forces. The cause of AC joint injury through direct means could result from contact with another player, the playing surface, or equipment. Conversely, an AC joint sprain may manifest through an indirect mechanism, such as when an athlete falls on an elbow or outstretched hand, thereby indirectly transmitting force to the AC joint [2]. It is reported that over 40% of all shoulder injuries may be attributed to AC joint injuries [3].

The mechanism of injury for an AC joint is falling on an outstretched arm and blunt trauma to the corner of the shoulder. Patients presenting with an AC joint injury commonly report experiencing pain in the anterosuperior region of the shoulder. In some cases, individuals with AC joint injuries may describe pain that extends to the neck or shoulder area, particularly exacerbated during activities involving cross-arm adduction. Physical examination may reveal signs such as swelling, bruising, or structural abnormalities in the AC joint, varying in presentation based on the extent of the injury. The tenderness is present over the AC joint as well. Due to pain, active and passive ROM is reduced. The "piano key sign" may be positive [4]. Post-AC joint injuries, non-surgical methods of treatment include rest, medication like anti-inflammatory drugs, local anesthetics, Kinesio tape, and various forms of physiotherapy exercises [5].

X-ray is a standard method for diagnosing AC injury or shoulder injuries. AC joint injury may not always diagnosed on X-rays with a lateral view and anteroposterior (AP), so X-rays are executed in the Zanca view. This view was taken in the bilateral anteroposterior (AP) view and cranial view by tilting the beam 10-15 degrees to compare the location of the joint with the opposite shoulder joint [6].

Rockwood stated that non-operative treatment is typically used for type I and II injuries while operative methods are employed for managing type IV and VI injuries [7]. Additionally, mobilization with movement (MWM) is a manual therapy technique in which high-velocity glides are applied to a peripheral joint to correct postural errors caused by injury or strain. It also reduces pain and improves patient outcomes [8].

Virtual reality (VR) is a new approach that elevates patient motivation and participation in training better than traditional rehabilitation. A virtual reality (VR) gaming software designed with a mechanism that generates VR limb movements. VR develops a significant amount of sensory and visual feedback while exercising [9].

## Case presentation

Patient information

An 18-year-old male, a state-level kabaddi athlete, presented with chief complaints such as pain in the left side of the shoulder. The patient was not able to perform any shoulder activity and was unable to sleep in the affected position. Before the complaint, he had the same two-time previous history of falls on his left shoulder while playing a kabaddi match. With this complaint, the patient went to orthopedics, where he was advised to conduct a radiographic investigation. After that, he underwent a radiological investigation, which revealed a grade II AC sprain. He was managed conservatively with medications and got relief in five days. After a week, he again started playing kabaddi, and the pain reoccurred. Then, he went to a private hospital, and he was advised and referred for further physiotherapy management.

Clinical findings

After obtaining written permission from the patient, an evaluation was conducted. The patient was fully conscious, cooperative, and well-oriented to time, place, and person. The patient was seated in an upright position, with left shoulder protraction, adduction, and external rotation. Pain on the anterior portion of his left shoulder was assessed using a numerical pain rating scale (NPRS), obtaining a score of 1/10 at resting and 6/10 during activities like cross-arm adduction movements and shoulder end-range abduction. The pain was described as dull and aching, but it had a sudden onset and gradually intensified. Palpation revealed grade 3 tenderness over the anterior portion of his left shoulder joint. Movement quality was assessed at grade 4, characterized by pain and incomplete ROM. The scarf test was positive.

Diagnostic assessment

**Figure 1:**
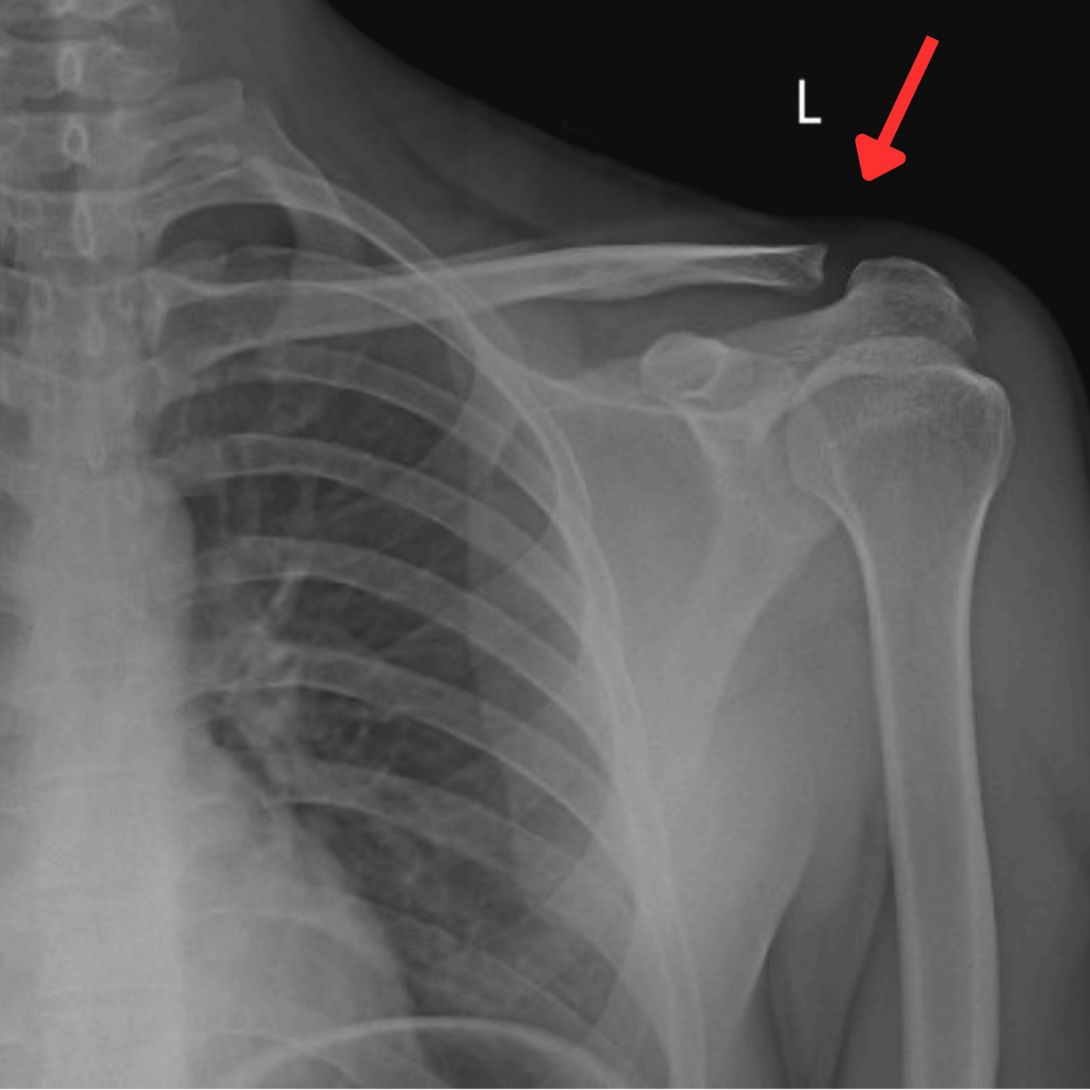
Radiograph of the left shoulder joint with grade II AC joint sprain AC: acromioclavicular

According to the Rockwood classification, radiological investigation in AP view of his left shoulder revealed a grade II AC joint sprain, as shown in Figure 1.

Clinical pre-intervention and post-intervention assessments of range of motion are described in Table 1.

**Table 1:** Shows pre-intervention and post-intervention goniometry for ROM of the shoulder joint ROM: range of motion

Shoulder Movements	Pre-intervention		Post-intervention	
	Left	Right	Left	Right
Flexion	0-160^0^	0-180^0^	0-175^0^	0-180^0^
Extension	0-50^0^	0-50^0^	0-50^0^	0-50^0^
Abduction	0-115^0^	0-170^0^	0-150^0^	0-170^0^
Horizontal adduction	0-70^0^	0-100^0^	0-90^0^	0-100^0^
Internal rotation	0-40^0^	0-65^0^	0-55^0^	0-65^0^
External rotation	0-50^0^	0-90^0^	0-70^0^	0-90^0^

Clinical pre-intervention and post-intervention assessment of manual muscle testing are described in Table 2.

**Table 2:** Shows pre-intervention and post-intervention manual muscle testing

Shoulder Muscles	Pre-intervention		Post-intervention	
	Left Side	Right Side	Left side	Right Side
Flexors	4/5	5/5	5/5	5/5
Extensors	3/5	5/5	4/5	5/5
Abductors	4/5	5/5	5/5	5/5
Horizontal Adductors	2/5	5/5	4/5	5/5
Internal rotators	4/5	5/5	5/5	5/5
External rotators	4/5	5/5	5/5	5/5

Physiotherapy intervention

**Figure 2:**
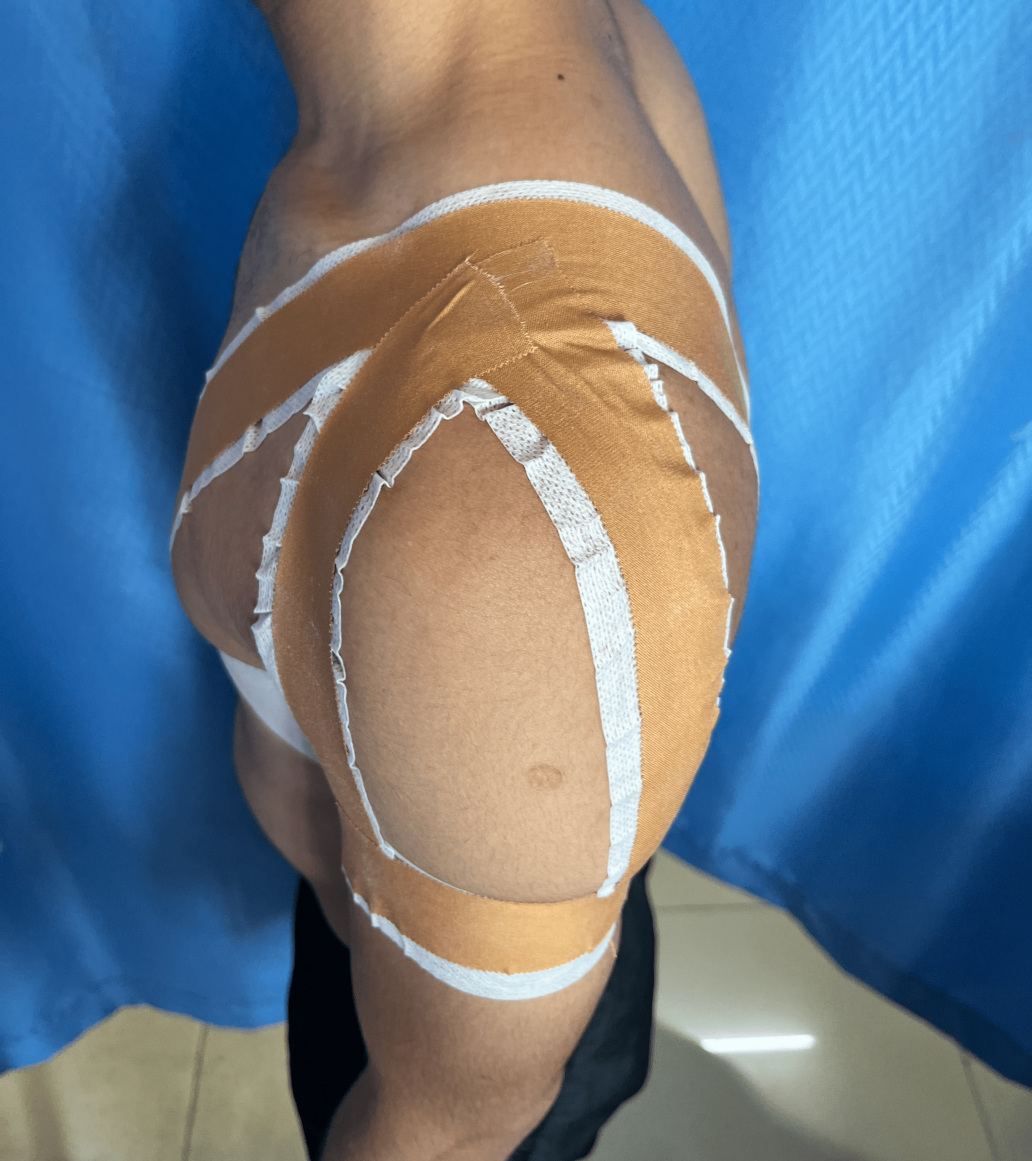
Rigid taping for the AC joint AC: acromioclavicular

**Figure 3:**
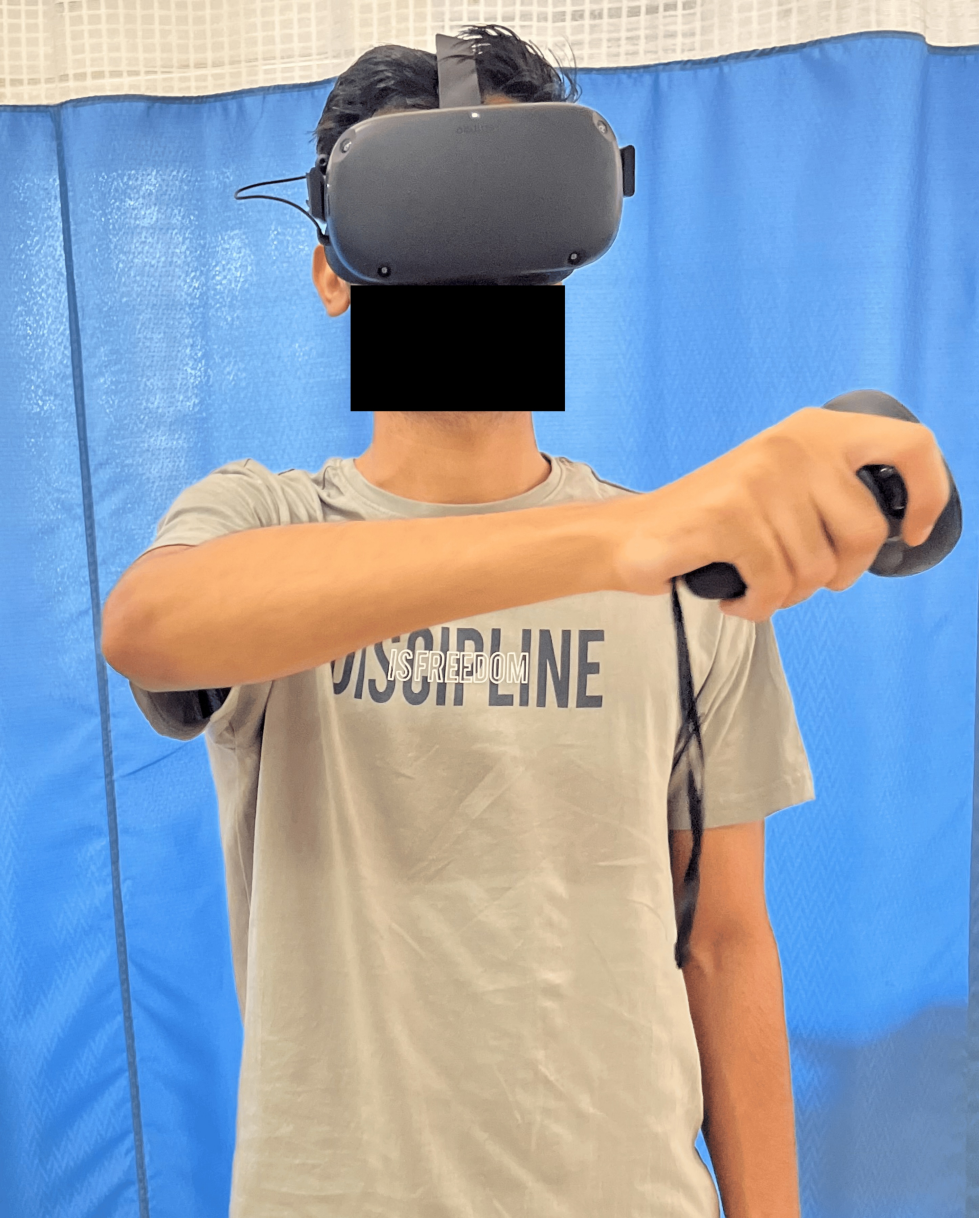
Patient performing VR-based rehabilitation for the AC joint VR: virtual reality; AC: acromioclavicular

**Figure 4:**
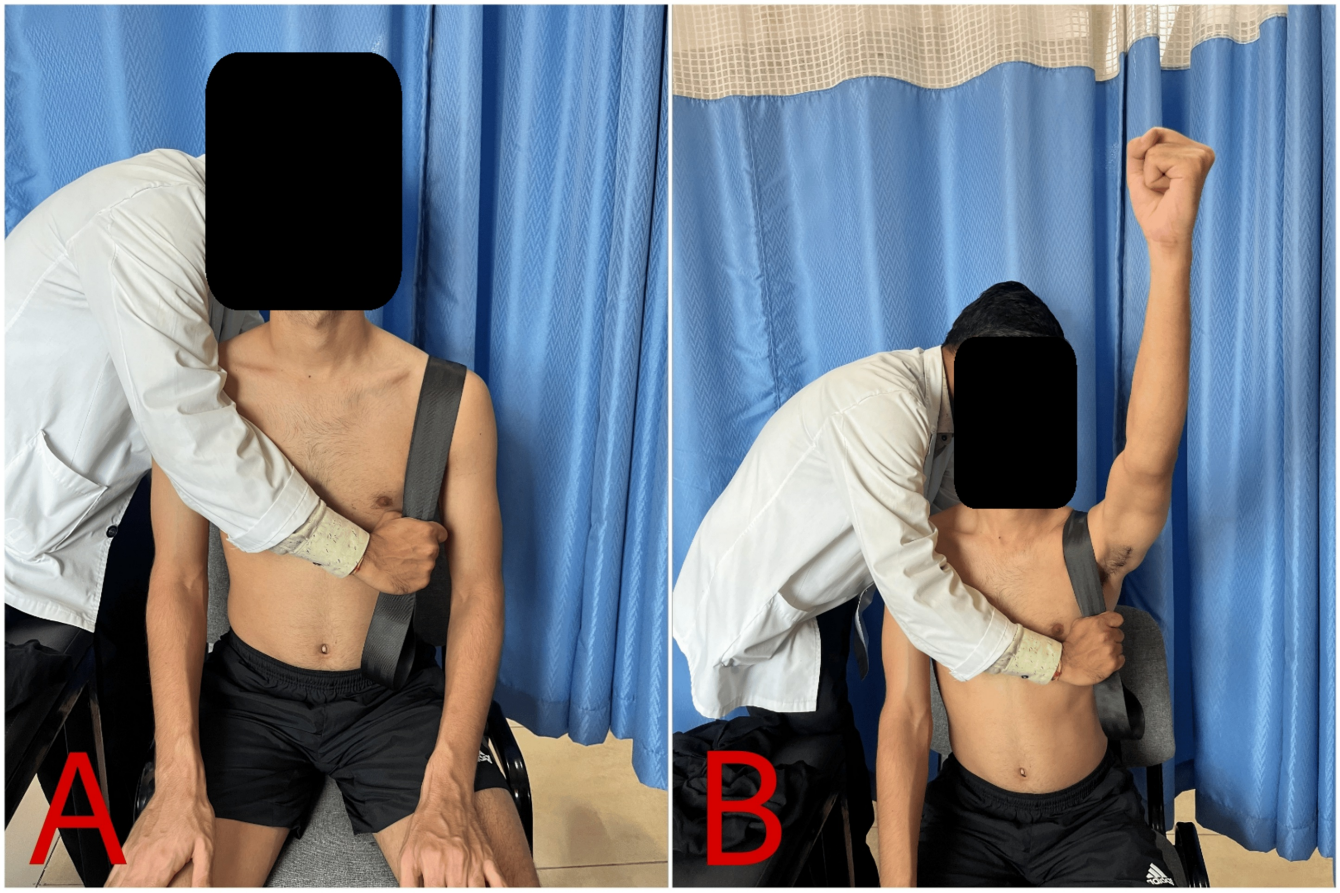
Mulligan's movement with mobilization for the AC joint AC: acromioclavicular

Table 3 depicts the physiotherapy intervention used for the patient. Figure 2 shows rigid taping for the AC joint. Figure 3 shows the patient performing VR-based rehabilitation for the AC joint, and Figure 4 shows Mulligan's MWM for the AC joint.

**Table 3:** Shows the physiotherapy intervention AC: acromioclavicular; ROM: range of motion; VR: virtual reality

Phase	Goal	Intervention	Dosage
Week I	Educate the patient	Motivating for rehabilitation explain their injury and what to do or don’t do as follows: Avoid hanging, avoid lifting heavy weights, avoid lying on the left side of the shoulder	Educate the patient about the health condition
	Pain reduction and minimized swelling	Cryotherapy: apply ice on painful and swollen areas and Ultrasound therapy	2-3 sessions for 10 minutes per day
	Correcting positional fault at the AC joint	Mulligan movement with mobilization	For 1 week
	Improve stability of the acromioclavicular joint.	By taping using rigid tape	Change every second day
Week II to III	To improve and maintain range of motion	Pain-free active shoulder flexion, extension, horizontal adduction, abduction ROM exercises, and pain-free shoulder stretching includes the posterior and anterior cuff, Meta Quest 2 virtual reality game - Ninja Fruit	15 repetitions, 3 sets
Week IV to V	To improve muscle strength	Shoulder isometric exercise includes static flexion, static extension, static internal rotation, and static external rotation	15 repetitions, 3 sets
Week VI	To improve and maintain muscle strength	Progress in muscle strengthening with Theraband exercises, including abduction, adduction flexion, extension, internal rotation, and external rotation	10 repetitions, 3 sets

Table 4 shows the intervention outcomes for the patient.

**Table 4:** Shows week one and week six scores of outcome measures NPRS: numerical pain rating scale; DASH: disabilities of the arm, shoulder, and hand; SPADI: shoulder pain and disability index

Scales	Week one	Week six
NPRS	NPRS score on rest is 2/10, NPRS score on activities like horizontal adduction and end-range abduction of the shoulder is 5/10	NPRS score on rest is 1/10, NPRS score on activities like horizontal adduction and end-range abduction of the shoulder is 1/10
DASH	60%	15%
SPADI	55%	10%

## Discussion

In our case report, the patient’s main concerns were pain over the anterosuperior site of the left shoulder, inability to perform shoulder activities, and inability to sleep on his left shoulder due to pain. The patient was diagnosed with a grade II acromioclavicular joint sprain and was advised physiotherapy treatment.

In 2022, Rahbar et al. discussed that the additional AC joint MWM with standard therapeutic exercises in an AC joint sprain has more impact in decreasing pain and disability and restoring ROM compared to therapeutic exercise rehabilitation alone [10]. In 2023, Longo et al. concluded that Meta Quest 2 (Meta Platforms, CA, USA) is a beneficial virtual reality system in patients with shoulder musculoskeletal disorders. VR rehabilitation could be comfortable, challenging, and motivating for patients toward rehabilitation [11]. In 2015, Satpute et al. discussed that the MWM technique showed an increase in shoulder internal rotation or ROM and a decrease in pain and disability after adding it to exercise along with hot fomentation when it was applied for three weeks of rehabilitation duration when compared to exercise and hot fomentation alone [12].

In the year 2023, Solemani et al. concluded in their systematic review and meta-analysis that adjunctive VR-based rehabilitation enhances upper limb motor recovery across multiple functional domains compared to conventional occupational therapy alone after stroke [13]. In 2023, Byers R et al. studied in their case report that the combination of AC joint mobilization with shoulder exercises for shoulder injuries was a perfect option for physiotherapy treatment in patients with AC joint sprain [14]. Nathani et al. 2024, in their study, concluded that multimodal approaches consisting of MWM, rigid taping, along with exercises proved to be effective in the case of an ACJ sprain [8].

Powell et al. (2022) demonstrated an effective method for estimating shoulder joint angles and torques in real-time during gamified exercises using a head-mounted display iVR system. This method uses only the controllers and headsets of intuitive gaming systems, making it ideal for at-home use because a therapist or expert does not need to be physically present. This has the potential to help therapists remotely evaluate patients and collect metrics that are often difficult to measure with limited two-dimensional videoconferencing. In conclusion, we can accurately provide evidence-based physical rehabilitation metrics using iVR systems paired with predictive models to redefine telehealth [15].

Carnevale et al. (2022) assessed the accuracy of the Oculus Quest 2 in measuring translational and rotational displacements within a range covering the whole values of interest for applications in shoulder rehabilitation [16].

One challenge in our study was ensuring consistent patient adherence to the VR rehabilitation protocol, which occasionally impacted treatment efficacy. Additionally, initial patient discomfort regarding the new kind of intervention like VR sometimes disrupted sessions for which proper education and familiarization were done. Despite using objective assessments, the reliance on subjective reports for certain outcomes suggests a need for more comprehensive measures in future studies. These limitations provide important context for interpreting our findings and underscore areas for improvement in VR rehabilitation research.

## Conclusions

After six weeks of rehabilitation, the patient’s active ROM in the shoulder was nearly at the normal range. The pain subsided, and strength in the upper extremities was regained. Just after the sixth week of rehabilitation, outcomes suggested that the combination of virtual reality rehabilitation along with acromioclavicular joint movement with mobilization, rigid taping, and therapeutic exercise for an acromioclavicular grade II sprain was the appropriate option for the patient managed with physiotherapy. Now, the patient has resumed playing and all other activities. The overall report concluded that our given rehabilitation had a positive impact on patient recovery.
